# Effect of Natural Chinese Herbal Supplements (TCMF4) on Lactation Performance and Serum Biomarkers in Peripartal Dairy Cows

**DOI:** 10.3389/fvets.2021.801418

**Published:** 2022-01-10

**Authors:** Yizhe Cui, Zhuorui Shan, Lintong Hou, Qiuju Wang, Juan J. Loor, Chuang Xu

**Affiliations:** ^1^College of Animal Science and Technology, Heilongjiang Bayi Agricultural University, Daqing, China; ^2^Heilongjiang Provincial Key Laboratory of Prevention and Control of Bovine Diseases, Heilongjiang Bayi Agricultural University, Daqing, China; ^3^Department of Animal Sciences, Division of Nutritional Sciences, University of Illinois, Urbana, IL, United States

**Keywords:** traditional Chinese herbal formula, additive, lactation performance, peripartal, cow

## Abstract

This study examined the effect of mixed medicinal herbs from China in the ground form on milk yield and various blood metabolites before and after parturition in Holstein cows. Crushed *Agastache rugosus, Scutellaria barbata, Pericarpium citri reticulate*, and *Radix glycyrrhizae* were used to develop TCMF4. Thirty-two Chinese Holstein cows were randomly divided into a control group or groups receiving 0.1, 0.3, or 0.5 kg TCMF4/cow/d from −7 through 21 d relative to parturition. Blood samples for serum isolation were collected at −7, −1, 1, 7, 14, and 21 d relative to parturition and used to measure glucose, β-hydroxybutyric acid (BHBA), total protein, albumin, globulin, and alkaline phosphatase. Milk production was recorded daily for the first 21 d postpartum, and composition was analyzed at 7, 14, and 21 d. Data were analyzed using a one-way analysis of variance (ANOVA) for multiple comparisons. The average milk production during the first 21-d postpartum was 28.7 ± 6.9, 27.2 ± 7.1, 31.2 ± 6.8, and 38.5 ± 6.1 kg/d for control group and groups receiving 0.1, 0.3, or 0.5 kg TCMF4. Thus, average daily milk production increased between 9 to 34% by supplementation with TCMF4 compared with the control group. Compared with the control group, in the middle dose group, milk concentrations of lactose and total protein decreased by 21 and 19%, respectively, at d 7 around parturition, while total solids increased by 23% at d 21 in the high-dose group. Furthermore, compared with the control group, serum BHBA decreased by 50 and 20% at d −1 and 21 around parturition in the high-dose group. Overall, TCMF4 supplementation improved dry matter intake (DMI) and milk production of dairy cows during the periparturient period without adverse effects on liver function, and plasma BHBA concentrations of dairy cows tended to decrease when dietary TCMF4 increased, which suggested that TCMF4 might be used as potential additives in dairy cows to improve production performance.

## Introduction

Chinese herbal feed additives represent a natural, multifunctional, and non-toxic approach to reducing disease incidence in dairy cows without the inherent risks that antibiotic use pose. These additives come mainly from plants' roots, stems, and leaves and have natural biological activities. There are enzymes in the body that transform the molecules in these plants into forms that are more easily used by tissues (1). Available data indicate that these additives aid in disease resistance, fattening and weight gain, and feed utilization efficiency in addition to shortening the feeding cycle, hence, providing safety and economic benefits.

Bioactive components in traditional Chinese medicines (TCM) can modulate microbial community function and enhance growth and *in vitro* nutrient digestibility (2) in sheep (3), goats (4), beef cattle (5), and cows (5). Among the TCM, *Pericarpium citri reticulatae* has been used widely in clinical practice to treat nausea, vomiting, and indigestion (6), while *Radix glycyrrhizae* has protective effects against the damage caused by both heat and oxidative stress (7). In addition to the above TCM, *Radix glycyrrhizae* has also been used in dairy cows (8).

The TCM comprises numerous species with many functions and is often used in a formula to obtain synergistic effects or to diminish possible adverse reactions (9). Accordingly, the primary function of the formula may be different depending on its composition. This study examined the effect of mixed medicinal herbs from China in the ground form on milk yield and various blood metabolites before and after parturition in Holstein cows. Four traditional Chinese herbal medicine, including crushed *Agastache rugosus, Scutellaria barbata, Pericarpium citri reticulatae*, and *Radix glycyrrhizae* were mixed to develop a TCM additive termed TCMF4.

Using the TCMF4 additive, our laboratory demonstrated a beneficial role in preventing metabolic disorders in Institute of Cancer Research (ICR) mice fed a high-fat diet (HFD) *in vivo*. It is unknown whether the additive can produce similar effects in the peripartum period when cows are most susceptible to liver-related metabolic disorders. Therefore, the specific objective of the present study was to generate milk production and plasma data as a function of increasing doses of TCMF4 supplementation around parturition in a commercial farm setting. These data would be valuable in evaluating the potential application of this feed additive in the dairy industry.

## Materials and Methods

The present study was conducted at the YuDa Dairy Farm (Heilongjiang, China). Holstein cows were used as experimental subjects in this study. All procedures were conducted using protocols approved by the Heilongjiang Provincial Key Laboratory of Prevention and Control of Bovine Diseases (Heilongjiang, China). All cows used in the current study were managed according to Heilongjiang Provincial Key Laboratory of Prevention and Control of Bovine Diseases (Heilongjiang, China), Heilongjiang Bayi Agricultural University (Heilongjiang, China). Cows were housed in individual tie-stalls and milked daily at 4:00, 10:00, and 16:00 a.m. Throughout the entire experimental period, the health of the cows was monitored and recorded.

### Animals, Diets, and Experimental Design

Thirty-two multiparous peripartal dairy cows of similar age (4, 5), parity (2, 3), similar milk production in last season (17 ± 1.3 kg/d), expected calving time with single calf (day 267–270 post insemination), no different BCS (3.5) and body weight (572.81 ± 35.26 kg), and clinical health were randomly assigned to a control diet (*n* = 8) or the control plus TCMF4 supplementation 0.1, 0.3, or 0.5 kg TCMF4/head/d from −7 through 21 d around parturition (The Chinese Veterinary Pharmacopoeia). The experimental groups were fed with corresponding doses of TCMF4 from −7 through 21 days. All cows received their corresponding diets as a total mixed ratio (TMR). The composition and nutrient contents of the feed ingredients are presented in Supplementary Tables 1, 2. All cows had free access to water throughout the entire experiment. The *Agastache rugosus, Scutellaria barbata, Pericarpium citri reticulatae*, and *Radix glycyrrhizae* used in this experiment were selected from the Chengdu Lotus Pond Chinese herbal medicine market (Sichuan, China). Moreover, in our previous study, chromatographic detection and safety evaluation was carried out on these Chinese medicines' components, which confirmed that these traditional Chinese plant medicines were non-toxic and harmless (10).

According to previous research, the plants mentioned above were ground into a 100-mesh fine powder with a grinder and mixed in a 25:25:25:25 ratio (10). The supplement was top-dressed on the TMR. In every morning the TMR mixed with TCMF4 for each day was fed to cows based on the dose group firstly, then the morning feeding TMR followed.

### Milk and Milk Component Yields

Milk yield was recorded at 0400, 1,000, and 1,600 daily after calving. The average across a 3-d collection period was used for Statistical Analysis. Milk components were measured at 7, 14, and 21 days around parturition using pooled samples, taking into account milk yield in the morning, midday, and evening. Samples were stored at 4°C until subsequent testing. To minimize the number of times samples were sitting at barn temperature, they were retrieved from the sampling device every 4 h and transferred to a refrigerator for storage at 4°C. Milk protein, fat, and lactose were measured the following day via an automatic milk analyzer (Delta Instruments, C330, HP, Netherlands).

### Blood Sampling and Analyses

Blood was collected from the coccygeal artery at −7, −1, 1, 7, 14, and 21 d relative to parturition. Samples were centrifuged at 3,500 rpm for 10 min, and the obtained sera were transferred into cryovials and stored at −20°C until the data were analyzed. Glucose (GLU) (ml076792) and β-hydroxybutyric acid (BHBA) (ml021780) were assessed in a Cobas C 501 analyzer UV spectrophotometer (Roche Diagnostics, Mannheim, Germany) using a commercially available kit. Total protein (TP) (ml060630), albumin (ALB) (ml076981), globulin (GLB) (ml063706), and alkaline phosphatase (ALP) (ml076536) contents were determined in a BT1500 automated biochemical analyzer (Biotecnica Instruments S.p.A., Roma, Italy) using commercially available kits (mlbio Biotechnology Co., Ltd., Shanghai, China).

### Statistical Analysis

All data were analyzed using the appropriate statistical analysis methods with SPSS (Statistical Package for the Social Sciences) 19.0 software (SPSS Incorporated, Chicago, IL, USA) and GraphPad Prism version 5 software. Data were tested for normality and homoscedasticity using the Shapiro-Wilk and Levene tests, respectively. For data with a Gaussian distribution, repeated measures analysis was performed for multiple comparisons with Bonferroni correction for data meeting homogeneity of variance or with Tamhane's T2 Analysis for data with heteroscedasticity. Data are expressed as the means ± SEM. Treatment differences were determined by the LSD (least significant difference) and were considered significant if *P* < 0.05. Significant differences in all figures are related to total and not related to specific time.

## Results

### Milk Yield

**Figure 1:**
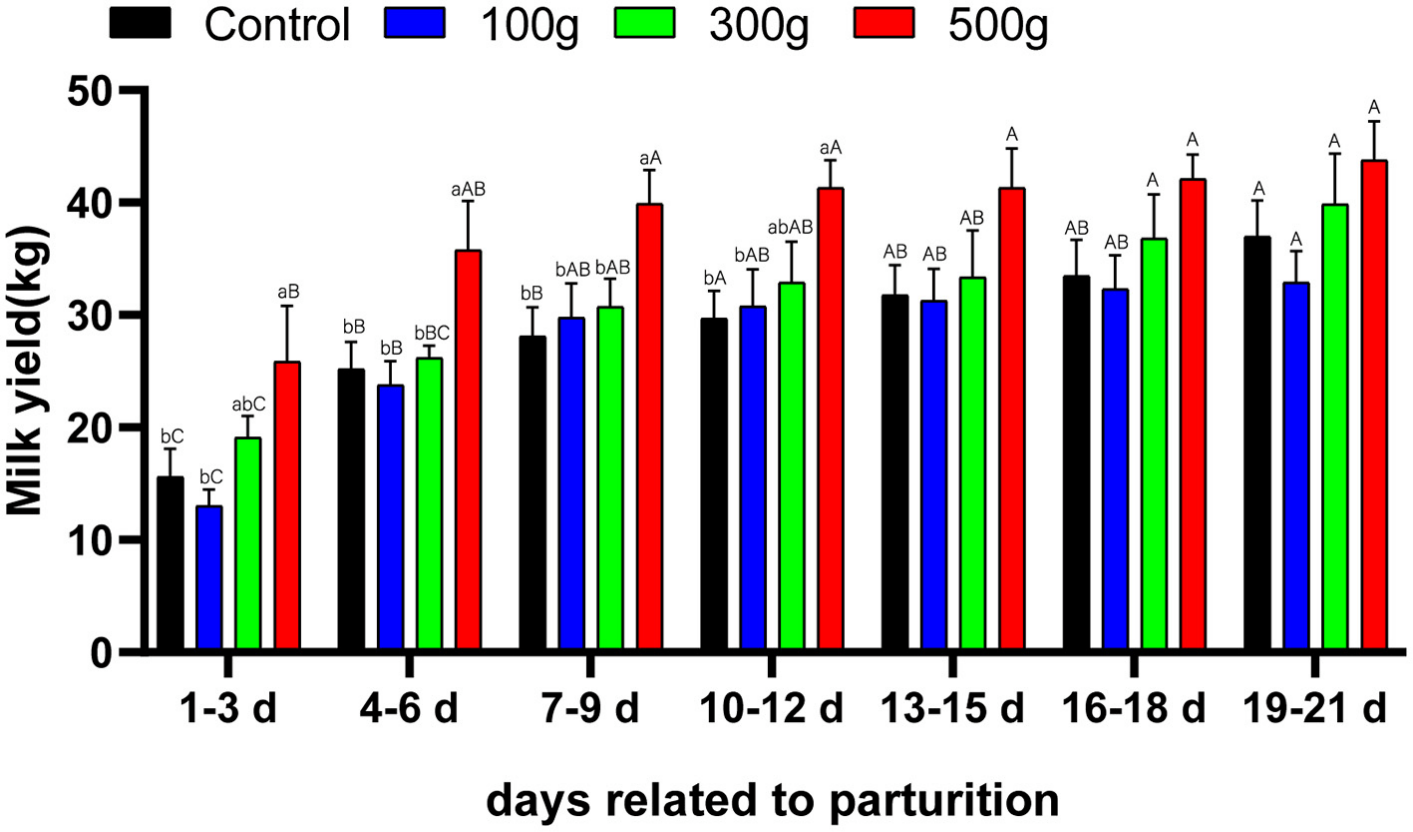
Effects of dietary TCMF4 on milk production in peripartal dairy cows. Milk was collected during the 3-d metabolic measurements for each period. Daily averages are represented. The control group was fed a basic diet: TCMF4 groups (control diet supplemented with TCMF4). ^a,b^Different superscripts within a row represent significant differences between groups (*P* < 0.05). ^A,B^Different superscripts in the rows represent significant differences in time (*P* < 0.05).

Compared with the control group, the milk yield of the low- and medium-dose groups was not significant (*P* > 0.05). Milk production of the high-dose group was significantly different from the control (*P* < 0.05) on days 1–3, 7–9, and 10–12, but there was no significant difference between the high-dose group on d 13–21 after calving (*P* > 0.05). Milk production of the high-dose group was significantly different from that of the low-dose (*P* < 0.05) and medium-dose group (*P* < 0.05) at 7–9 and 10–12 days. Increasing TCMF4 supplementation led to gradual increases in milk yield (Figure 1).

### DMI

**Figure 2:**
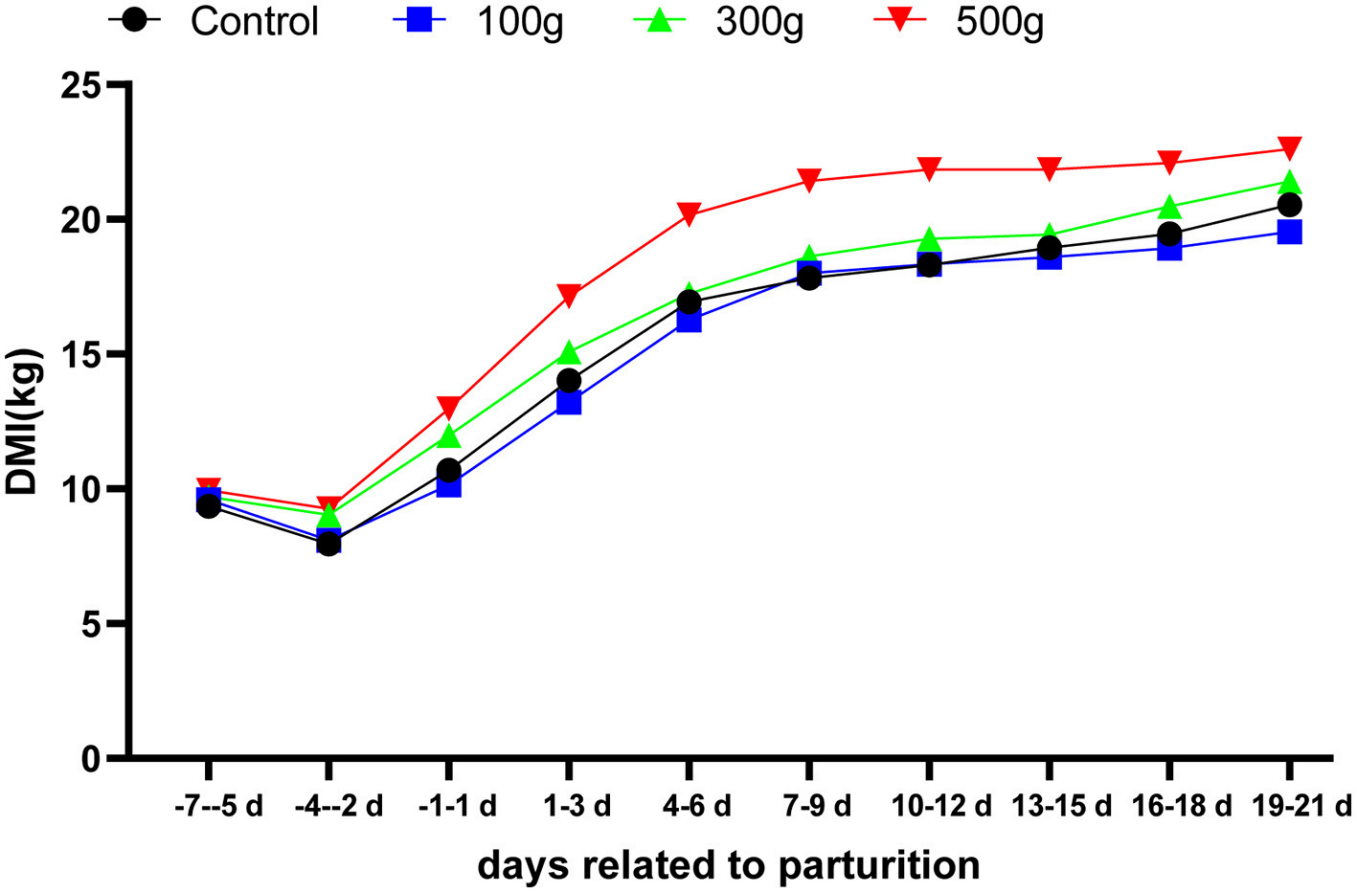
Effects of dietary TCMF4 on DMI in peripartal dairy cows. DMI was collected during the 3-d metabolic measurements for each period. Daily averages are represented. The control group was fed an essential diet: TCMF4 groups (control diet supplemented with TCMF4).

There was a period of decline in DMI during the prepartum period, with the high- and medium-dose groups declining more gently than the control and low-dose groups during days −7 to −1. Compared with the control and low-dose groups, DMI of the high-dose groups significantly increased (*P* < 0.05) on days 1–12. DMI of the high-dose group was significantly different from the control (*P* < 0.05) on days 1–21, but there was no significant difference between the TCMF4 group on d 13–21 after calving (*P* > 0.05) (Figure 2).

### Milk Composition

**Figure 3:**
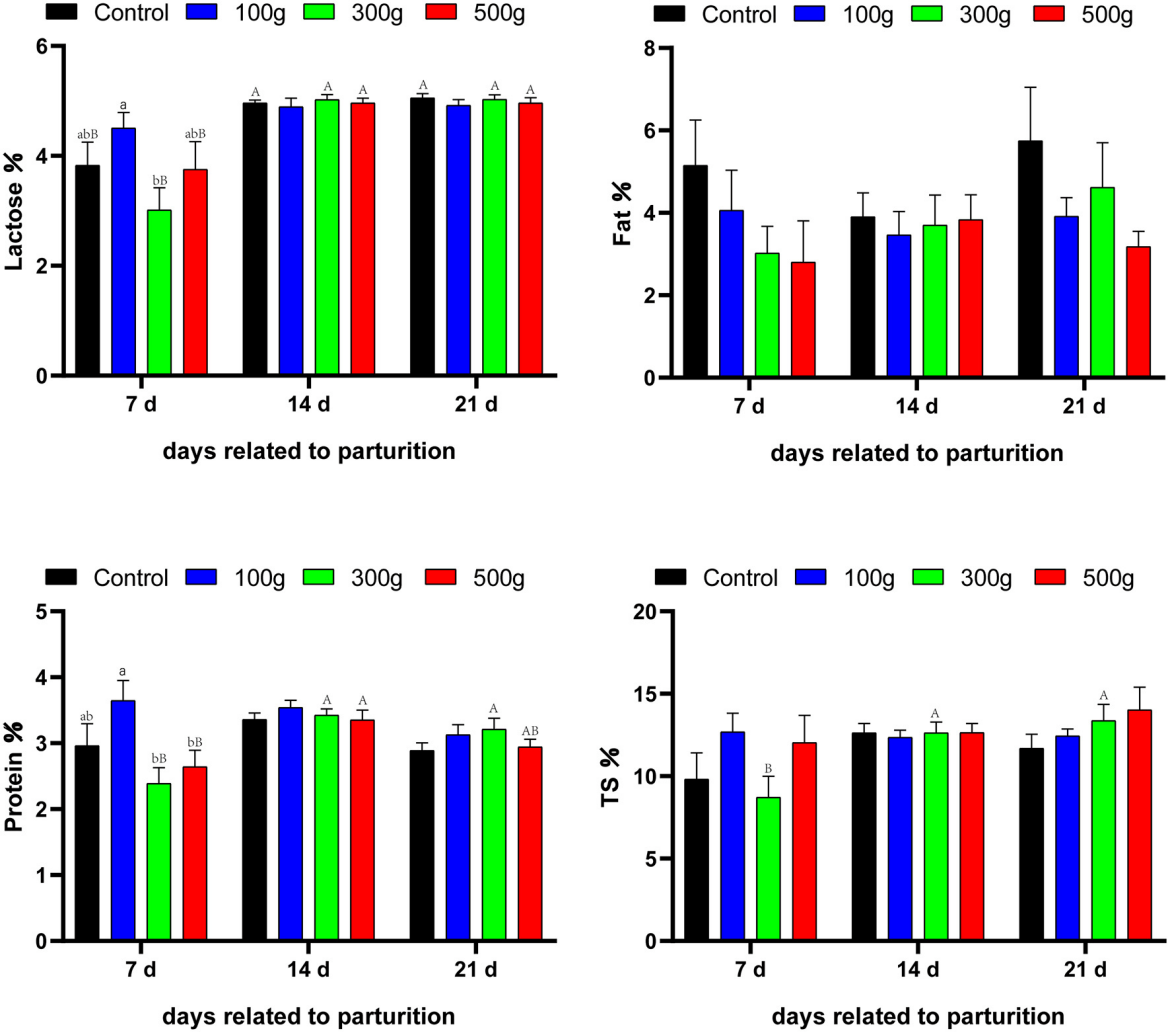
Effects of dietary TCMF4 on milk composition in peripartal dairy cows. The milk components from the experimental cows were measured on day 7 before calving, day 1 before calving, and days 7, 14, and 21 after delivery. The control group was fed a basic diet; TCMF4 groups (control diet supplemented with TCMF4). ^a,b^Different superscripts within a row represent significant differences between groups (*P* < 0.05). ^A,B^Different superscripts in the rows represent significant differences in time (*P* < 0.05).

The lactose content of the middle-dose group was significantly lower than that of the low-dose group at 7 d postpartum (*P* < 0.05). There was no significant difference in lactose content between the groups at other time points (*P* > 0.05). The milk fat content was not significantly (*P* > 0.05) among difference group. There was no significant difference in milk fat content at other time points (*P* > 0.05). The milk protein content of the low-dose group was significantly higher than that of the middle-dose and high-dose groups at 7 d postpartum (*P* < 0.05). There was no significant difference in milk protein content at other time points (*P* > 0.05). Milk TS content of the medium-dose group at 21 d was significantly higher than that of the medium-dose group at 7 d (*P* < 0.05) (Figure 3).

### Serum Biomarkers During the Periparturient Period

**Figure 4:**
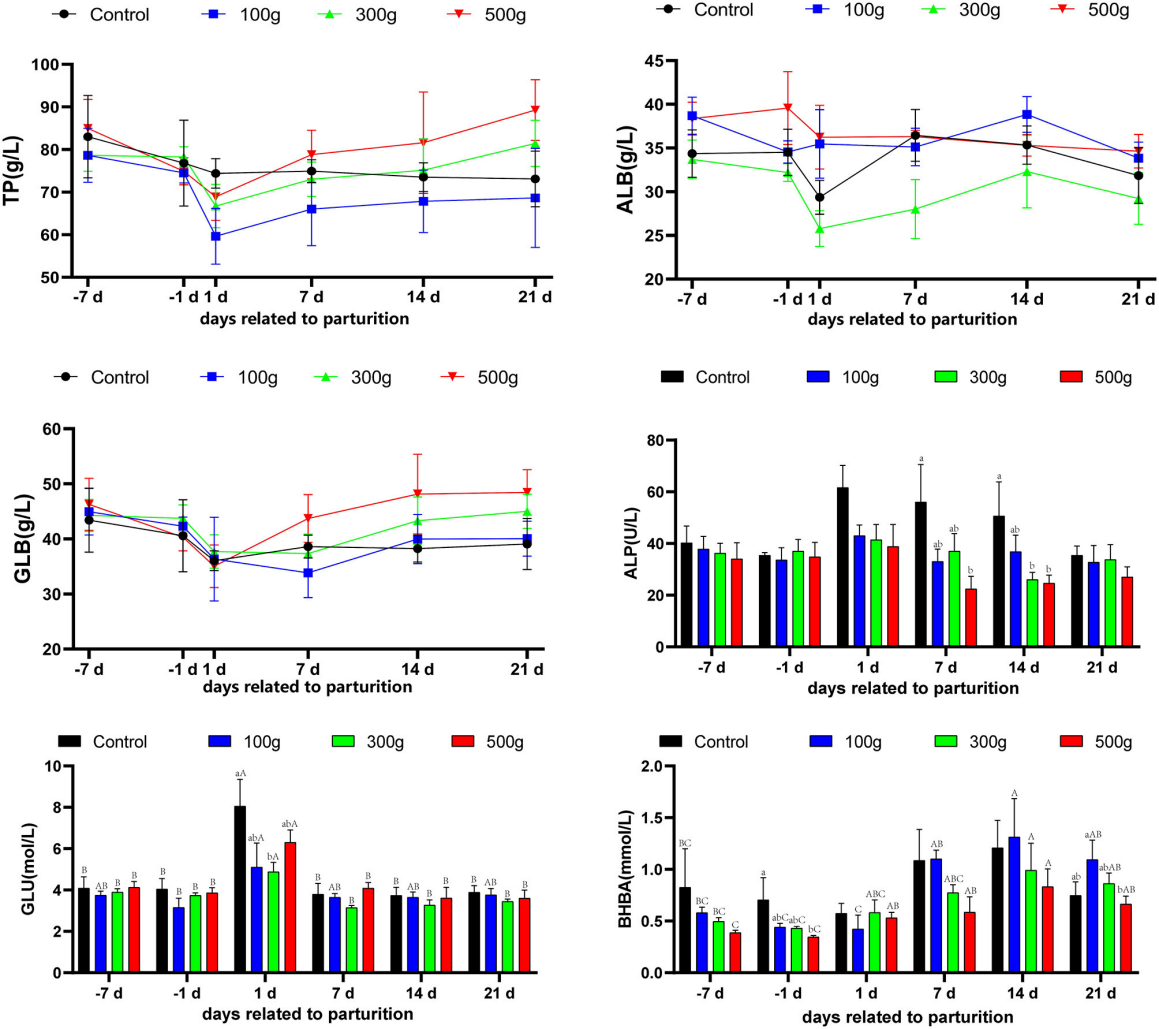
Effects of TCMF4 on serum indexes of cows during the periparturient period. TP, total protein; ALB, albumin; GLB, globulin; ALP, alkaline phosphatase; GLU, glucose; BHBA, β-hydroxybutyric acid. The milk components of the experimental cows were measured on the day of delivery and the 7th, 14th day, and 21st days after delivery. The control group was fed a basic diet: TCMF4 groups (control diet supplemented with TCMF4). ^a,b^Different superscripts within a row represent significant differences between groups (*P* < 0.05). ^A,B^Different superscripts in the rows represent significant differences in time (*P* < 0.05).

The change of serum TP concentration in the control group was gradually decreased over time; while Serum TP concentration in the three groups of TCMF4 was gradually decreased from 7 d before to 1 d after parturition, and the lowest on the day of parturition (Figure 4), and gradually increased from days 1–21, and the TP concentration increased with the increase of TCMF4 concentration. The change of ALB can be seen by the control group is unstable, and with the increasing concentration of TCMF4, ALB also tends to be more stable, especially in the high dose group, which is at a relatively high and stable level. The profile of GLB in each group was similar to that of TP concentration, being lowest on the day of parturition and gradually increasing from day 1 to 21, with increasing concentrations of TCMF4. ALP activity ranged from high to low between 7 and 1 day before calving; it rose to the highest level on the day of calving and then decreased. Compared to the control group, the three TCMF4 groups were more stable; the ALP activity was also significantly lower in the high and medium dose groups on the day of calving and on postpartum day 14 (*P* < 0.05). Serum BHBA was very unstable in the control group, and serum BHBA in the high-dose group was significantly lower than the control group (*P* < 0.05) on the first day before calving, and at 21 days postpartum, it was significantly lower compared with the low-dose group (*P* < 0.05). BHBA decreased with increasing TCMF4 concentrations and gradually increased instability.

## Discussion

The peripartal period is the most critical stage in the life cycle of dairy cows (11). Cows must go through the process of pregnancy, delivery, and lactation, which causes physiological stress. In addition, dry matter intake (DMI) decreases, and the demand for nutrients by the fetus or mammary gland increases, leading to a negative energy balance. These events affect dairy cows' immune capacity and postpartum functional recovery (12). Chinese herbal medicine formulations could be an important practical tool to help alleviate these adverse effects. For instance, Chinese herbal medicine formulations improved nutrient utilization, growth, and quality of livestock products and elicited biological effects at the tissue level (13). Among the additives selected in the current study are four traditional Chinese herbal medicines, including crushed *Agastache rugosus, Scutellaria barbata, Pericarpium citri reticulatae*, and *Radix glycyrrhizae* were mixed to develop a TCM additive termed TCMF4. *Pericarpium citri reticulatae* has beneficial and protective effects on the liver (14). At the same time, *Agastache rugosus* modified rumen fermentation ecology by feed degradability, microbial enzyme activities, and total protozoa counts (15). *Radix glycyrrhizae* invigorates the spleen and replenishes Qi (16). There are many polysaccharides, volatile oils, tannins, alkaloids, and organic acids in *Agastache rugosus, Scutellaria barbata, Pericarpium citri reticulatae*, and *Radix glycyrrhizae*. Some of these compounds can provide a source of energy, while others can elicit physiologic responses that could enhance feed efficiency for milk production (17).

### Effects of TCMF4 on Milk Production Performance

According to the Theory of Traditional Chinese Medicine, a deficiency in Qi (18) and blood often occurs before and after parturition; thus, a key objective for improving milk yield is to replenish the Qi and blood (19). Hence, a feed additive such as The TCMF4 formulated based on traditional plant components could invigorate Qi and blood and elicit a positive effect (20) (directly or indirectly) on milk yield.

Orange peel silage was evaluated the effect of replacing corn silage of multiparous lactating Holstein cows, and the results showed that orange peel silage as a substitute for corn silage for feeding dairy cows did not show adverse changes in the rumen environment and showed promising results in the increase of fat in milk of Holstein cows (21). Licorice root supplementation was used on dairy cows, and results showed a positive role of licorice in modifying cow cheeses' chemical and physical properties, reducing lipid oxidation, and inducing changes in color and flavor with a presumable improvement in consumer acceptability (22).

In the present study, the decreasing trend in DMI from day −7 to −1 was consistent with previous reports (10), with a more moderate decrease in DMI with dose. Day 1–21 showed an increasing DMI with dose, while milk yield in the 500 g group increased by more than 30%, indicating that TCMF4 could improve the palatability of TMR and thus increase the DMI to influence the increase of milk yield. Another possible explanation for the positive effect of TCMF4 on milk production might have been associated with its content of bioactive and other nutrients (23). Flavonoids can improve the permeability of blood vessels, reduce blood lipid and cholesterol (24), and elicit the same effect as phytoestrogens (25). For instance, phytoestrogens can protect against zearalenone-induced reproductive toxicity in male mice (26) and improve ovarian and uterine function (27). Phenolic compounds have antioxidant capacity and their inhibitory effects on α-glucosidase, pancreatic lipase and hyaluronidase (28). The composition of essential oil is complex, mainly small molecules of aldehydes and esters, monoterpenes, sesquiterpenes and small molecules of aromatic compounds, etc. (29). Essential oil has anti-inflammatory, anti-allergic, anti-microbial, anti-mutagenic and anti-cancer and anthelmintic effects (30).

Herbal galactagogues include fenugreek, blessed thistle, milk thistle, fennel, anise, and nettle, which could effectively elicit a positive effect on milk synthesis (31). The addition of plant extracts relieved negative effects of energy at peak lactation of sheep (32). Herbal extracts are likely to improve subclinical ketosis in dairy cows (33). Together, previous data suggest that the inclusion of TCMF4 as a dietary feed supplement in animal production could be effective in health and production performance.

### Effects of TCMF4 on Plasma Parameters

While the studies' gradual decrease in serum TP and ALB confirmed previous data (34). Changes in TP and ALB content are related to liver function and energy metabolism. Once the liver cells are damaged, the synthesis of proteins is reduced (35). The decrease of ALB concentration in each group in the prepartal period might have been due to impaired liver function (36), the acceleration of ALB catabolism (37), and the dilution of ALB concentration by differences in blood volume (38). The gradual increase of TP and the stabilization of ALB in the three groups of TCMF4 after parturition can indicate that TCMF4 can better protect liver function after parturition and make the liver status of postpartum cows more important stable.

The level of BHBA in the postpartum period was higher than that in the antepartum period, which agrees with a previous report (39). The increased energy demand for milk production along with decreased DMI (40) leads to intense lipolysis, subsequently resulting in elevated circulating levels of non-esterified fatty acid (NEFA) and BHBA (41). Thus, lower serum BHBA concentration in the high-dose group postpartum in this study indicated that TCMF4 could ameliorate negative energy balance, ensuing in a lower extent of body fat mobilization.

Peak lactation is characterized by enhanced lipid mobilization and decreased blood GLU (42). GLU is the primary substrate and oxidative fuel for lactose and milk protein synthesis; moreover, GLU is mainly derived from liver gluconeogenesis in dairy cows (43). However, dramatically decreased DMI and increased milk yield intensify body fat mobilization, which leads to ketosis, fatty liver, and other metabolic disorders (44). Except for being utilized by animals as nutrients, TCM contributes to enhancing animal immune function due to their abundant beneficial factors such as polysaccharides, alkaloids, and essential oils (45), which might partly explain the positive effect of TCMF4 supplementation on production performance during the periparturient period in this study.

## Conclusions

Overall, TCMF4 supplementation improved dry matter intake and milk production of dairy cows during the periparturient period without adverse effects on liver function, and plasma BHBA concentrations of dairy cows tended to decrease when dietary TCMF4 increased, which suggested that TCMF4 might be used as potential additives in dairy cows to improve production performance.

## Data Availability Statement

The original contributions presented in the study are included in the article/Supplementary Material, further inquiries can be directed to the corresponding authors.

## Ethics Statement

The animal study was reviewed and approved by Heilongjiang Provincial Key Laboratory of Prevention and Control of Bovine Diseases (Heilongjiang, China) and Heilongjiang Bayi Agricultural University (Heilongjiang, China). Written informed consent was obtained from the owners for the participation of their animals in this study.

## Author Contributions

YC, QW, LH, and CX put forward the idea and concept of this paper in the beginning. YC and LH conceived and designed the experimental procedure. YC and QW prepared the original draft. JL reviewed with a critical review. ZS participated in method development and validation. JL and CX participated in study design and coordination and helped draft the manuscript. All authors have read and approved the final manuscript.

## Funding

The work was supported in part by the Academician Cooperation Project of Heilongjiang Provence Science and Technology Program (YS20B04), Heilongjiang Province postdoctoral research start-up fund (LBH-Q20161), Development of Local Universities, and Heilongjiang Bayi Agricultural University (ZRCQC201803 and ZRCLG201904).

## Conflict of Interest

The authors declare that the research was conducted in the absence of any commercial or financial relationships that could be construed as a potential conflict of interest.

## Publisher's Note

All claims expressed in this article are solely those of the authors and do not necessarily represent those of their affiliated organizations, or those of the publisher, the editors and the reviewers. Any product that may be evaluated in this article, or claim that may be made by its manufacturer, is not guaranteed or endorsed by the publisher.
