# Supplementary material for: Effect of Natural Chinese Herbal Supplements (TCMF4) on Lactation Performance and Serum Biomarkers in Peripartal Dairy Cows
Source: Front Vet Sci. 2022 Jan 10;8:801418. doi: 10.3389/fvets.2021.801418 (PMC8784967; doi:10.3389/fvets.2021.801418)
Supplement: Supplementary file 1 [file Data_Sheet_1.docx]

Table S1. Formula composition and nutritional value of the basic diet in early calving (%)

| Ingredient | Value | Nutrient composition | Value |
| --- | --- | --- | --- |
| *Leymus chinensis* | 14.5 | Net energy for lactating (MJ/kg) | 8.23 |
| Maize Silage | 60.7 | Crude protein | 16.94 |
| Soybean meal | 2.8 | Crude fiber | 38.86 |
| Cottonseed meal | 2.5 | Calcium | 1.75 |
| Rapeseed meal | 2.0 | Phosphorus | 0.96 |
| Brewers' dried grains | 2.9 |  |  |
| Wheat bran | 1.2 |  |  |
| Premix^1^ | 1.4 |  |  |

^1^Provided per kilogram of premix: 200000 IU vitamin A; 45000 IU vitamin; 1500 mg vitamin E; 1550 mg nicotinic acid; 400 mg Cu; 600 mg Mn; 2150 mg Zn; 34800 mg Mg; 20 mg Se; 18 mg Co.

Table S2. Formula composition and nutritional value of the basic diet in the postpartum period

| Ingredient | Value | Nutrient composition | Value |
| --- | --- | --- | --- |
| *Leymus chinensis* | 15 | Net energy for lactating (MJ/kg) | 11.4 |
| Maize Silage | 35 | Crude protein | 20.4 |
| Alfalfa | 6 | Crude fiber | 35.81 |
| DDGS | 12.5 | Calcium | 1.35 |
| Corn | 9 | Phosphorus | 1.22 |
| Dry beer distillers' grains | 6.7 |  |  |
| Brewers' dried grains | 0.5 |  |  |
| Salt | 0.5 |  |  |
| Premix^1^ | 4.2 |  |  |

^1^Provided per kilogram of premix: 200000 IU vitamin A; 45000 IU vitamin; 1500 mg vitamin E; 1550 mg nicotinic acid; 400 mg Cu; 600 mg Mn; 2150 mg Zn; 34800 mg Mg; 20 mg Se; 18 mg Co.
